# Quantitative Boltzmann–Gibbs Principles via Orthogonal Polynomial Duality

**DOI:** 10.1007/s10955-018-2060-7

**Published:** 2018-05-10

**Authors:** Mario Ayala, Gioia Carinci, Frank Redig

**Affiliations:** Delft Institute of Applied Mathematics, Delft University of Technology, Mekelweg 4, 2628 CD Delft, The Netherlands

**Keywords:** Orthogonal polynomials, Duality, Boltzmann–Gibbs principle, Fluctuation field

## Abstract

We study fluctuation fields of orthogonal polynomials in the context of particle systems with duality. We thereby obtain a systematic orthogonal decomposition of the fluctuation fields of local functions, where the order of every term can be quantified. This implies a quantitative generalization of the Boltzmann–Gibbs principle. In the context of independent random walkers, we complete this program, including also fluctuation fields in non-stationary context (local equilibrium). For other interacting particle systems with duality such as the symmetric exclusion process, similar results can be obtained, under precise conditions on the *n* particle dynamics.

## Introduction

The Boltzmann–Gibbs principle is an important ingredient in the study of fluctuation fields of interacting particle systems [8]. It basically states that on the central limit scale, the fluctuation field of local functions can be replaced by a constant times the density fluctuation field, or in other words, it can be replaced by its projection on the one dimensional space generated by the density fluctuation field (where projection has to be understood in an appropriate Hilbert space of macroscopic quantities [1]). The aim of the present paper is to refine and quantify the Boltzmann–Gibbs principle in the context of particle systems with duality, using fluctuation fields of orthogonal polynomials. Indeed, it turns out that replacing the fluctuation field of a local function by its projection on the density field corresponds to the projection on the fluctuation fields of orthogonal polynomials of order one. Therefore, the Boltzmann–Gibbs principle easily follows from an estimation of the covariance of fluctuation fields of orthogonal polynomials of order two and higher. In this paper, for independent random walkers we quantify the precise order of these covariances of fluctuation fields of orthogonal (Charlier) polynomials of order *n* for all $n\in \mathbb {N}$, and therefore we are able to give an orthogonal decomposition of the fluctuation field of any local function, which is a generalization of the Boltzmann–Gibbs principle. Next, still in the context of independent random walkers, we are able to extend this result in a non-equilibrium setting, using the fact that product of Poisson measures are preserved under this dynamics, i.e., a strong form of propagation of local equilibrium holds in that context.

One of the basic ingredients of our approach is stochastic duality, a property shared by a certain class of interacting particle systems such as independent random walkers [2], exclusion process, inclusion process, brownian energy process, etc. (see [3] for a review on the subject). Thanks to duality the n-body correlation functions obey closed equations, not involving higher correlations. This has many implications, such as the possibility to study the decay properties of correlation functions [6] and to study small perturbation of the original process [4].

In this paper we exploit a duality property with orthogonal polynomials (see e.g. [11]) combined with precise estimates (of local limit type) of the *n* particle dynamics. Therefore, the results immediately apply in the context of the stationary symmetric exclusion process, and more generally for particle systems where these precise estimates (of local limit type) of the *n* particle dynamics can be obtained (e.g. via the log-Sobolev inequality [9]). Next we consider the orthogonal polynomial fluctuation fields themselves and prove that they converge in the sense of generalized processes, i.e., as a random space-time distribution. The rest of our paper is organized as follows: In Sect. 2 we formally introduce our system of random walkers, and the basic concepts and properties needed for the development of this paper. In Sect. 3, on the context of stationarity, we start by introducing our results for the simplest non-trivial example of second order and move to a generalization first to higher orders and in a next stage to more general functions. We present in Sect. 4 an extension of these last results to a non-equilibrium setting. Finally in Sect. 5 we show how under additional assumptions our results can be extended to other interacting particle systems.

## Basic Notions

### Independent Random Walkers

We consider a system of independent random walkers (IRW), an interacting particle system where particles randomly hop on the lattice $\mathbb {Z}^d$ without interaction and with no restrictions on the number of particles per site. Configurations are denoted by $\eta , \xi , \zeta$ and are elements of $\Omega =\mathbb {N}^{\mathbb {Z}^d}$ (where $\mathbb {N}$ denotes the natural numbers including zero). We denote by $\eta _x$ the number of particles at *x* in the configuration $\eta \in \Omega$. The generator working on local functions $f:\Omega \rightarrow \mathbb {R}$ is of the type

1 $$\begin{aligned} \mathscr {L}f(\eta ) = \sum _{i,j} p(i,j)\eta _i (f(\eta ^{ij})-f(\eta )) \end{aligned}$$

where $\eta ^{ij}$ denotes the configuration obtained from $\eta$ by removing a particle from *i* and putting it at *j*. Additionally, we assume that *p*(*i*, *j*) is a translation invariant, symmetric, irreducible Markov transition function on $\mathbb {Z}^d$, i.e.,

1. $$p(i,j)=p(j,i)= p(0,j-i)$$.
2. $$\sum _{j \in \mathbb {Z}^d} p(i,j)=1.$$
3. There exists $R>0$ such that $p(i,j)=0$ for $|i-j|>R$.
4. For all $x,y\in \mathbb {Z}^d$ there exist $i_1=x,\ldots ,i_n=y$ such that $\prod _{k=1}^n p(i_k,i_{k+1})>0$.

For the associated Markov process on $\Omega$, we use the notation $\{\eta (t):t\ge 0\}$, i.e., $\eta _x(t)$ denotes the number of particles at time *t* at location $x \in \mathbb {Z}^d$.

It is well known that these particle systems have a one parameter family of homogeneous (w.r.t. translations) reversible and ergodic product measures $\nu _{\bar{\rho }}, \rho >0$ with Poisson marginals

$$\begin{aligned} \nu _\rho (n)= \frac{\rho ^n}{n!} e^{-\rho } \end{aligned}$$

This family is indexed by the density of particles, i.e.,

$$\begin{aligned} \int \eta _0 d\nu _{\bar{\rho }}= \rho \end{aligned}$$

#### Remark 2.1

Notice that for these systems the initial configuration has to be chosen in a subset of configurations such that the process $\{\eta (t):t\ge 0\}$ is well-defined. A possible such subset is the set of tempered configurations. This is the set of configurations $\eta$ such that there exist $C, \beta \in \mathbb {R}$ that satisfy $|\eta (x)| \le C |x|^\beta$ for all $x\in \mathbb {R}^{d}$. We denote this set (with slight abuse of notation) still by $\Omega$, because we will always start the process from such configurations, and this set has $\nu _{\bar{\rho }}$ measure 1 for all $\rho$. Since we are working mostly in $L^2(\nu _{\bar{\rho }})$ spaces, this is not a restriction.

### Orthogonal Polynomial Self-duality

The self-duality of the process we introduced and which we need in the sequel is as follows. We denote by $\Omega _f$ the set of configurations with a finite number of particles (we denote by $\Vert \xi \Vert =\sum _x \xi _x$ this number of particles), and the self-duality function will then be a function $D:\Omega _f\times \Omega \rightarrow \mathbb {R}$ such that the following properties hold.

1. Self-duality:
  2 $$\begin{aligned} \mathbb {E}_\eta \big [D(\xi ,\eta _t)\big ]=\mathbb {E}_\xi \big [D(\xi _t, \eta )\big ] \end{aligned}$$
  for all $\xi \in \Omega _f, \eta \in \Omega$ (where we remind that $\eta \in \Omega$ is always chosen such that the process $\{\eta (t): t\ge 0\}$ is well-defined when starting from $\eta$).
2. Factorized polynomials:
  3 $$\begin{aligned} D(\xi ,\eta )=\prod _{i\in \mathbb {Z}^d} d(\xi _i,\eta _i) \end{aligned}$$
  where $d(0,n)=1$, and $d(k,\cdot )$ is a polynomial of degree *k*.
3. Orthogonality:
  4 $$\begin{aligned} \int D(\xi , \eta ) D(\xi ', \eta ) d\nu _{\bar{\rho }}(\eta ) = \delta _{\xi ,\xi '} a(\xi ) \end{aligned}$$
  where $$a(\xi )= \Vert D(\xi , \cdot )\Vert _{L^2(\nu _{\bar{\rho }})}^2$$.

Notice that these functions will depend on the parameter $\rho$, but we suppress this dependence in order not to overload notation.

The duality functions which, for independent random walkers, satisfy properties (2),(3) and (4) are known in the literature as Charlier polynomials. These polynomials can be expressed in terms of hypergeometric functions as follows:the single site duality functions $d \left( k, n \right)$ satisfy the three terms recurrence relation

5 $$\begin{aligned} d(k+1,n) = d(k,n) - \frac{n}{\rho } \; d(k,n-1) \end{aligned}$$

additionally to this recurrence relation, at least two more relations can be found.

#### Remark 2.2

To avoid minor confusions please notice that in [7] a relation between “classical” and new orthogonal duality polynomials is given. Where with classical polynomials we mean

6 $$\begin{aligned} d(k,n) = \frac{n!}{(n-k)!} \end{aligned}$$

and the way they relate is given by

7 $$\begin{aligned} D(\xi ,\eta ) = \prod _{x \in \mathbb {Z}^d} \sum _{j=0}^{\xi _x} {{\xi _x}\atopwithdelims (){j} } (-\rho )^{\xi _x-j} \frac{\eta _x!}{(\eta _x-j)!} \end{aligned}$$

However expression (7) differs by a factor $-\rho ^{|\xi |}$ from the traditional form of the Charlier polynomials found in the literature:

8 $$\begin{aligned} \tilde{D}(\xi ,\eta ) = \prod _{x \in \mathbb {Z}^d} \sum _{j=0}^{\xi _x} {{\xi _x}\atopwithdelims (){j} } (-\rho )^{-j} \frac{\eta _x!}{(\eta _x-j)!} \end{aligned}$$

The factor $-\rho ^{||\xi ||}$ is however invariant under the dynamics of our process that conserves the total number of particles $||\xi (t)||$, and hence its addition preserves the duality property. Duality function (8) is precisely the one that satisfies the relation given in (5) when starting with $d(0,n)=1$.

For more details on orthogonal duality and a proof of self-duality with respect to this function we refer to [7] and [11]. In those papers a more complete study is provided, which includes the case of other processes such as exclusion and inclusion, among others.

We denote by $p_t(\xi ,\xi ')$ the transition probability to go from the configuration $\xi$ to $\xi '$ in time *t*. A key ingredient for our proof of the Boltzmann–Gibbs principle and its extensions is the following elementary consequence of duality with orthogonal duality functions.

#### Lemma 2.1

Let $$\xi ,\xi '\in \Omega _f$$, then

9 $$\begin{aligned} \int \mathbb {E}_\eta (D(\xi ,\eta _t)) D(\xi ',\eta ) d\nu _{\bar{\rho }}(\eta )= p_t(\xi ,\xi ') a(\xi ') \end{aligned}$$

#### Proof

We use self-duality to compute

$$\begin{aligned} \int \mathbb {E}_\eta [D(\xi ,\eta _t)] D(\xi ',\eta ) d\nu _{\bar{\rho }}(\eta )= & {} \int \mathbb {E}_\xi [D(\xi _t,\eta )] D(\xi ',\eta ) d\nu _{\bar{\rho }}(\eta ) \\= & {} \sum _{\zeta } p_t(\xi ,\zeta ) \int D(\zeta ,\eta ) D(\xi ',\eta ) d\nu _{\bar{\rho }}(\eta ) \\= & {} p_t(\xi ,\xi ') a(\xi ') \end{aligned}$$

that proves the result. $\square$

#### Remark 2.3

Notice that (9) in particular implies that if $\eta _0$ is initially distributed according to $\nu _\rho$ then

10 $$\begin{aligned} \text {Cov}_{\nu _{\bar{\rho }}} \left( D(\xi ,\eta _t) D(\xi ',\eta ) \right) \ge 0 \end{aligned}$$

i.e. duality orthogonal polynomials are positively correlated.

Lemma 2.1 provides a big simplification since it allows to transfer most of the uncertainty of our process to the transition kernel $p_t(\xi ,\xi ')$ of two configurations in $\Omega _f$. Here $\{\xi (t), t\ge 0\}$ is a Markov process with countable state space, conserving only $\Vert \xi (t)\Vert$ in the course of time, and then easier to treat. In the Appendix we provide an estimate of this kernel by means of the local limit theorem.

### Fluctuation Fields

Let ${\mathscr {S}}(\mathbb {R}^d)$ be the set of Schwarz functions on $\mathbb {R}^d$, and denote by ${\mathscr {S}}'(\mathbb {R}^d)$ the corresponding distributions space. Moreover we denote by $\tau _x$ the spatial shift, i.e., $\tau _x (\eta )_y= \eta _{y+x}$,. Fix $\varphi \in {\mathscr {S}}(\mathbb {R}^d)$ and let $f:\Omega \rightarrow \mathbb {R}$ be a local function, we define its fluctuation field on scale *N* as

11 $$\begin{aligned} \mathsf {X_N}(f,\eta ;\varphi ):=a_N(f)\sum _{x\in \mathbb {Z}^d} \varphi \left( \tfrac{x}{N}\right) (\tau _x f(\eta )-\psi _f(\rho )) \end{aligned}$$

where

12 $$\begin{aligned} \psi _f(\rho ):=\int f d\nu _{\bar{\rho }}, \qquad \tau _x f(\eta ):= f(\tau _x\eta ) \end{aligned}$$

and $a_N(\cdot )$ is a suitable normalization constant depending on *f*. The field $\mathsf {X_N}(f,\eta ;\cdot )$ is a Schwarz-distribution associated to the configuration $\eta$. An important case is the density fluctuation field, where we chose $f(\eta )=\eta _0$, $a_N(f)={N^{-d/2}}$.

The time-dependent fluctuation field at scale *N* is then defined as

13 $$\begin{aligned} {\mathsf {X_N}}(f,t;\varphi )= \mathsf {X_N}(f,\eta (N^2t);\varphi ) \end{aligned}$$

the diffusive rescaling anticipates the natural macroscopic time-scale in this symmetric process, which has the linear heat equation as hydrodynamic limit. $\{ {\mathsf {X_N}}(f,t;\cdot ), \; t\ge 0\}$ is then a Schwarz-distribution valued stochastic process.

### Boltzmann–Gibbs Principle

The Boltzmann–Gibbs principle makes rigorous the idea that the density fluctuation field is the fundamental fluctuation field, because the density is the only (non-trivial) conserved quantity in the process under consideration. This means that one can replace, in first approximation, the fluctuation field of a function *f* by its “projection on the density field”. For a local function *f* this projection is the fluctuation field of the function $P_1(f):=\psi _f'(\rho ) (\eta _0-\rho )$, where $\psi _f(\rho ) = \int f d \nu _{\bar{\rho }}$.

The standard statement of the Boltzmann–Gibbs principle is given in the following theorem.

#### Theorem 2.1

For all *f* local, and $\varphi \in {\mathscr {S}}(\mathbb {R}^d)$ and for all $T>0$

14 $$\begin{aligned} \lim _{N\rightarrow \infty }\mathbb {E}_{\nu _{\bar{\rho }}} \left[ \left( \frac{1}{N^{d/2}} \int _0^T\left( \mathbf{\mathsf {X_N}}(f,t;\varphi )- \mathbf{\mathsf {X_N}}(P_1(f),t;\varphi )\right) dt \right) ^2\right] =0. \end{aligned}$$

We refer to [8] for the proof of Theorem and for a comprehensive discussion of the result that is valid in a more general context and not only for the process considered in the present paper.

### Fluctuation Fields of Orthogonal Polynomials

For $n\in \mathbb {N}$ we denote by ${\mathscr {H}}_n$ the (real) Hilbert spaces generated by the polynomials $D(\xi ,\cdot )$ with degree at most *n*, i.e. $||\xi ||\le n$. We have of course the inclusion ${\mathscr {H}}_0=\mathbb {R}\subset {\mathscr {H}}_1\subset {\mathscr {H}}_2\subset \ldots$ and the union of the spaces ${\mathscr {H}}_n$ is dense in $L^2(\nu _\rho )$. Moreover, for every $f\in L^2(\nu _\rho )$ its projection on ${\mathscr {H}}_n$ is given by

15 $$\begin{aligned} f_n= \sum _{\xi \in \Omega _f: \Vert \xi \Vert \le n} \langle f, D(\xi ,\cdot )\rangle \frac{D(\xi ,\cdot )}{a(\xi )} \end{aligned}$$

where $\langle \cdot ,\cdot \rangle$ denotes the $L^2 (\nu _{\bar{\rho }})$ inner product.

The aim of what follows is to show that the Boltzmann–Gibbs principle is an instance of a more general statement concerning the fluctuation behavior of functions which are orthogonal to ${\mathscr {H}}_n$ for some $n\in \mathbb {N}$. This is (in some sense to be explained below) the case for the function $f-P_1(f)$.

For $\xi \in \Omega _f$, $\varphi \in {\mathscr {S}}(\mathbb {R}^d)$ we define the n-th order polynomial fluctuation field as

16 $$\begin{aligned} X_N(\xi ,\eta ,\varphi )&:=\sum _{x\in \mathbb {Z}^d}\varphi \left( \tfrac{x}{N}\right) \, D( \xi , \tau _x \eta ) \nonumber \\&=\sum _{x\in \mathbb {Z}^d}\varphi \left( \tfrac{x}{N}\right) \, D(\tau _x \xi , \eta ). \end{aligned}$$

## Stationary Case

### Second Order Polynomial Field

We start with the simplest non-trivial example for independent random walkers started from a product measure with homogeneous Poisson marginals. To illustrate our point let us start with a simple computation, which contains all the important ingredients of the more general Theorem 3.1 below. Consider the field

17 $$\begin{aligned} X_N^{(2)}(\eta ;\varphi ):=X_N(2\delta _0,\eta ,\varphi )=\sum _{x \in \mathbb {Z}^d}\varphi \left( \tfrac{x}{N}\right) \, D(2\delta _x, \eta ) \end{aligned}$$

The notation $X_N^{(2)}$ suggests that this is in some sense the ”second order” polynomial field. In the orthogonal polynomial language, this is the field of the second order Charlier polynomial:

18 $$\begin{aligned} D(2\delta _x, \eta )=\eta _x(\eta _x-1)-2\rho (\eta _x-\rho )-\rho ^2 \end{aligned}$$

recall from earlier that

$$\begin{aligned} a ( 2 \delta _0) = \int (D(2\delta _x,\eta ))^2 d\nu _\rho (\eta ) \end{aligned}$$

then we have the following.

#### Proposition 3.1

The second order polynomial field $X_N^{(2)}(\eta ;\varphi )$ is such that

1. For $t>0$ we have
  19 $$\begin{aligned} \mathbb {E}_{\nu _{\bar{\rho }}}\left[ X_N^{(2)}(\eta (t);\varphi )\, X_N^{(2)}(\eta (0);\varphi )\right] =a ( 2 \delta _0) \,\sum _{x,y \in \mathbb {Z}^d}\varphi (\tfrac{x}{N})\varphi (\tfrac{y}{N}) (p_t(x,y))^2 \end{aligned}$$
2. As a consequence, for $t>0$ we have
  20 $$\begin{aligned} \lim _{N\rightarrow \infty }\mathbb {E}_{\nu _{\bar{\rho }}}\left[ X_N^{(2)}(\eta (N^ 2t);\varphi ) X_N^{(2)}(\eta (0);\varphi )\right] = \frac{d\cdot a ( 2 \delta _0) }{(2\pi t)^d}\int _{\mathbb {R}^{2d}} e^{-\frac{d|x-y|^2}{t}} \varphi (x)\varphi (y) dx dy \end{aligned}$$

#### Proof

The first statement follows from self-duality and Lemma 2.1. For the second statement we use that $\varphi$ has compact support, call this support *S*, and define

21 $$\begin{aligned} M := \max \{ d(x,y) : x, y \in S\} \end{aligned}$$

it follows from Theorem 6.2 that there exists $c= c(M)$ such that

$$\begin{aligned} \sup _{x:|x|\le M N \sqrt{t}} p^{RW}_{N^2t} (x) \le \bar{p}_{N^2t}(x) \left( 1+ \frac{c}{ N\sqrt{t}}\right) \end{aligned}$$

with $\bar{p}_{t}(\cdot )$ as defined in (71). Then from (28) it follows that

$$\begin{aligned}&\mathbb {E}_{\nu _{\bar{\rho }}}\left[ X_N^{(2)}(\eta (t);\varphi )X_N^{(2)}(\eta (0);\varphi )\right] \\&\qquad \qquad = a ( 2 \delta _0) \sum _{x,y \in S}\varphi (\tfrac{x}{N})\varphi (\tfrac{y}{N})\,\bar{p}_{N^2t}(x) \bar{p}_{N^2t}(y) \left( 1+ \frac{c}{ N\sqrt{t}}\right) ^2 \\&\qquad \qquad = a ( 2 \delta _0) \cdot \frac{d}{(2\pi t)^{d}}\cdot \frac{1}{N^{2d}}\sum _{x,y \in S}\varphi (\tfrac{x}{N})\varphi (\tfrac{y}{N}) e^{-\frac{d(z-y)^2}{tN^2}} \left( 1+ \frac{c}{ N\sqrt{t}}\right) ^2 \end{aligned}$$

and letting $N \rightarrow \infty$ we obtain the r.h.s. of (20). $\square$

In the current context the Boltzmann–Gibbs principle for the fluctuation field of the function $f = \eta _0 ( \eta _0 -1)$ is a consequence of Proposition 3.1. We make this statement more transparent with the following corollary

#### Corollary 3.1

The field $X_N^{(2)}(\eta (N^ 2 t);\varphi )$ is such that for all $T>0$ and for all *N* big enough

22 $$\begin{aligned} \frac{1}{N^d}\int _0^T\int _0^T\mathbb {E}_{\nu _{\bar{\rho }}}\left[ X_N^{(2)}(\eta (N^ 2 t);\varphi ) X_N^{(2)}(\eta (N^2 s);\varphi )\right] \, ds \, dt\le C(T) N^{-\frac{2d}{ 2 + d}} \end{aligned}$$

More precisely, (20) gives a better estimate of the order of the covariance of the fluctuation field in the diffusive time-scale as $N\rightarrow \infty$.

#### Proof

Given the fact that the RHS of (20) has an indetermination at $t=0$. Hence we derive the following estimate for the integrand in (22)

$$\begin{aligned}&\frac{1}{N^d}\mathbb {E}_{\nu _{\bar{\rho }}}\left[ X_N^{(2)}(\eta (N^ 2 t);\varphi ) X_N^{(2)}(\eta (N^2 s);\varphi )\right] \\&\qquad \qquad =\,K_\rho \, \frac{1}{N^d} \sum _{x \in \mathbb {Z}^d}\varphi (\tfrac{x}{N}) p_{N^2(t-s)}(x,y) \sum _{y \in \mathbb {Z}^d} \varphi (\tfrac{y}{N}) p_{N^2(t-s)}(x,y) \\&\qquad \qquad \le \,K_{\rho } p_{N^2(t-s)}(0,0) \Vert \varphi \Vert _1 \mathbb {E}_x \varphi (\tfrac{X_t}{N}) \\&\qquad \qquad \le \,K_{\rho } p_{N^2(t-s)}(0,0) \Vert \varphi \Vert _1 \Vert \varphi \Vert _{\infty } \end{aligned}$$

at this point we could have concluded (22) by naively estimating $p_{N^2(t-s)}(0,0)$ by one. Nevertheless our aim is to provide a more quantitative statement. Hence, we distinguished the cases $|t-s| \ge \epsilon _N$ and $|t-s| < \epsilon _N$ where $\epsilon _N$ is to be optimized. By the LCLT

23 $$\begin{aligned} p_{N^2(t-s)}(0,0) \le \frac{d}{(2\pi N^2(t-s))^{d/2}} \end{aligned}$$

then

24 $$\begin{aligned} p_{N^2(t-s)}(0,0) \le {\left\{ \begin{array}{ll} \frac{d}{N^d \epsilon _N^{d/2}}, &{} \text {if}\ |t-s| \ge \epsilon _N \\ 1 &{} \text {if}\ |t-s| < \epsilon _N \end{array}\right. } \end{aligned}$$

Hence the integral is bounded by

25 $$\begin{aligned}&\int _0^T\int _0^T \frac{1}{N^d}\mathbb {E}_{\nu _\rho }\left[ X_N^{(2)}(\eta (N^ 2 t);\varphi ) X_N^{(2)}(\eta (N^2 s);\varphi )\right] \, ds \, dt \nonumber \\&\qquad \qquad \qquad \le K_{\rho } \Vert \varphi \Vert _1 \Vert \varphi \Vert _{\infty } \frac{T^2}{2} \left[ \frac{d}{N^d \epsilon _N^{d/2}} + d \epsilon _N \right] \end{aligned}$$

Assume $\epsilon _N$ is of the form $N^{-\alpha }$, optimality then comes from solving for $\alpha$

$$\begin{aligned} N^{-\alpha } = N^{-d} N^{d/2 \alpha } \end{aligned}$$

after elementary computations we find $\alpha = \frac{2d}{d+2}$. Which in fact not only shows that the Boltzmann–Gibbs principle holds, but also provides us with a better estimate of the order of convergence. $\square$

Back to the second order polynomial fluctuation fields, and for the sake of transparency, we make explicit the dependency on the “coordinate points” $$x_1, x_2 $$ and redefine the fields in terms of the orthogonal duality polynomials as follows:

26 $$\begin{aligned} X_N^{(2)}(x_1, x_2,\eta ;\varphi ):=\sum _{x \in \mathbb {Z}^d}\varphi \Big (\tfrac{x}{N}\Big ) D(\delta _{x_1+x}+\delta _{x_2+x} , \eta ) \end{aligned}$$

Notice then, that in Proposition 3.1 we treated for $x_1=x_2=0$. It is necessary then to verify that Proposition 3.1 is not only result of this particular choice we made, consider then for $x_1\not =x_2$ the field

27 $$\begin{aligned} X_N^{(2),\not =}(x_1, x_2,\eta ,\varphi )= \sum _{x \in \mathbb {Z}^d} \varphi (\tfrac{x}{N})(\eta _{x+x_1}-\rho )(\eta _{x+x_2}-\rho ) \end{aligned}$$

where the upper index $\not =$ refers to the fact that $x_1\not = x_2$. We then have the following analogous of Proposition 3.1.

#### Proposition 3.2

The second order polynomial fluctuation field $X_N^{(2),\not =}(x_1, x_2,\eta ;\varphi )$ is such that

1. For $t>0$ we have
  28 $$\begin{aligned}&\mathbb {E}_{\nu _{\bar{\rho }}}(X_N^{(2),\not =}(x_1, x_2,\eta (t);\varphi )X_N^{(2),\not =}(x_1, x_2,\eta (0);\varphi )) \nonumber \\&\qquad \qquad = a( \delta _{x_1} + \delta _{x_2})\sum _{x,y \in \mathbb {Z}^d}\varphi \left( \tfrac{x}{N}\right) \varphi \left( \tfrac{y}{N}\right) p_t(x+x_1,x+x_2;y+x_1,y+x_2) \nonumber \\&\qquad \qquad \qquad +\,a( \delta _{x_1} + \delta _{x_2})\sum _{x,y \in \mathbb {Z}^d}\varphi \left( \tfrac{x}{N}\right) \varphi \left( \tfrac{y}{N}\right) p_t(x+x_1,x+x_2;y+x_2,y+x_1)\nonumber \\ \end{aligned}$$
2. As a consequence, for $t>0$ we have
  29 $$\begin{aligned}&\lim _{N\rightarrow \infty } \mathbb {E}_{\nu _{\bar{\rho }}}\left( X_N^{(2),\not =}(x_1, x_2,\eta (N^ 2t);\varphi ) X_N^{(2),\not =}(x_1, x_2,\eta (0);\varphi )\right) \nonumber \\&\qquad \qquad =\frac{2 a( \delta _{x_1} + \delta _{x_2}) d}{(2\pi t)^d}\int _{\mathbb {R}^{2d}} e^{-\frac{d|x-y|^2}{t}} \varphi (x)\varphi (y) dx dy. \end{aligned}$$

#### Proof

The argument for the first statement is similar to the one in the proof of Proposition 3.1, the difference is that now

$$\begin{aligned} D(\delta _{x+x_1} +\delta _{x+x_2},\eta )= (\eta _{x+x_1}-\rho )(\eta _{x+x_2}-\rho ) \end{aligned}$$

is the product of two first order Charlier polynomials, which by the assumption of factorized polynomials allows us to proceed in the same way than before. Furthermore, in this case we have

30 $$\begin{aligned}&p_t(\delta _{x+x_1}+ \delta _{x+x_2},\delta _{y+x_1}+ \delta _{y+x_2} ) \nonumber \\&\qquad \qquad = p_t(x+x_1,x+x_2;y+x_1,y+x_2) + p_t(x+x_1,x+x_2;y+x_2,y+x_1)\nonumber \\ \end{aligned}$$

which is the source of the second term in (28). In the second statement is necessary to verify that $x_1$ and $x_2$ do not play a role in the leading order

31 $$\begin{aligned}&\mathbb {E}_{\nu _{\bar{\rho }}}(X_N^{(2),\not =}(x_1, x_2,\eta (N^ 2t);\varphi ) X_N^{(2),\not =}(x_1, x_2,\eta (0);\varphi )) \nonumber \\&\qquad \qquad =a( \delta _{x_1} + \delta _{x_2})\sum _{x,y \in \mathbb {Z}^d}\varphi (\tfrac{x}{N})\varphi (\tfrac{y}{N}) p_{N^2t}(x+x_1,x+x_2;y+x_1,y+x_2) \nonumber \\&\qquad \qquad \qquad +\,a( \delta _{x_1} + \delta _{x_2})\sum _{x,y \in \mathbb {Z}^d}\varphi (\tfrac{x}{N})\varphi (\tfrac{y}{N}) p_{N^2t}(x+x_1,x+x_2;y+x_2,y+x_1)\nonumber \\ \end{aligned}$$

The first term in the RHS of (31) can be treated in the same way than before. For the second term, we just have to notice

$$\begin{aligned} |x+x_1-y-x_2|^2 +|x+x_2-y-x_1|^2 = 2|x-y|^2 +2|x_1-x_2|^2 \end{aligned}$$

and proceed in the same way. $\square$

Now we show how to generalize this result and discuss the case of higher order fields.

### Higher Order Fields

Let $k\in \mathbb {N}$ and denote by $\mathbf {x}\in \mathbb {Z}^{kd}$ the coordinates vector $\mathbf {x}:=(x_1,\ldots ,x_k)$, with $x_i\in {\mathbb {Z}}^d$, $i=1,\ldots , k$. We denote by $\xi (\mathbf {x})$ the configuration associated to $\mathbf {x}$, i.e. $\xi _x(\mathbf {x})=\sum _{i=1}^k\mathbf {1}_{x=x_i}$. We define $||\mathbf {x}||:=||\xi (\mathbf {x})||=k$. Here $x_i$ is the position of the *i*-th particle, where particles are labeled in such a way that the dynamics is symmetric. For a more extensive explanation of the labeled dynamics we refer the reader to [5]. We denote by $\hat{\tau }_z$, $z\in \mathbb {Z}^d$ the shift operator acting on the coordinate representation:

32 $$\begin{aligned} \hat{\tau }_z \mathbf {x}= (z+x_1, \ldots , z+x_k), \qquad \text {and then} \qquad \tau _z\xi = \xi (\hat{\tau }_z \mathbf {x}) \end{aligned}$$

Because of the translation invariance of the dynamics we have that

33 $$\begin{aligned} p_t(\xi (\hat{\tau }_y \mathbf {x}), \xi (\hat{\tau }_z \mathbf {x}))=p_t(\xi (\mathbf {x}), \xi (\hat{\tau }_{z-y} \mathbf {x})) \end{aligned}$$

With an abuse of notation, we keep denoting by $p_t(\mathbf {x}, \mathbf {y})$ the transition probability of the labeled particles in the coordinate representation.

#### Remark 3.1

The relation between the transition probabilities in the coordinate and in the configuration representations is given by

34 $$\begin{aligned} p_t(\xi (\mathbf {x}),\xi (\mathbf {y}))=\sum _{\mathbf {x}': \xi (\mathbf {x}')=\xi (\mathbf {y})} p_t(\mathbf {x}, \mathbf {x}') \end{aligned}$$

Notice that it is presicely from relation (34) that a factor of 2 appears in Proposition 3.2 and not in Proposition 3.1. We can expect that in this general setting the difference among cases will become more cumbersome. To avoid any further notational difficulties we introduce the following:

Let $\mathscr {P}_k$ be the set of permutations of $\{1,\ldots , k\}$, for $\sigma ,\sigma ' \in \mathscr {P}_k$ we define the following equivalence relation:

35 $$\begin{aligned} \sigma \sim \sigma ' \quad \text {mod} \quad \mathbf {x} \qquad \text {iff} \quad x_{\sigma (i)}=x_{\sigma '(i)} \quad \forall i \in \{1,\ldots ,k\} \end{aligned}$$

and define $\mathscr {P}_k(\mathbf {x}):=\mathscr {P}_k/\sim _{\mathbf {x}}$. Then we have

36 $$\begin{aligned} |\mathscr {P}_k(\mathbf {x})|=\frac{k!}{\prod _{i\in \mathbb {Z}^d}\xi _i(\mathbf {x})!} \end{aligned}$$

For each $\sigma \in \mathscr {P}_k(\mathbf {x})$ we define the new coordinate vector $\mathbf {x}^{(\sigma )}$ such that

37 $$\begin{aligned} \mathbf {x}^{(\sigma )}_i= x_{\sigma (i)} \end{aligned}$$

thus we can write

38 $$\begin{aligned} p_t(\xi (\mathbf {x}), \xi (\hat{\tau }_{z} \mathbf {x}))=\sum _{\mathbf {x}': \xi (\mathbf {x}')=\xi (\hat{\tau }_z \mathbf {x})} p_t(\mathbf {x}, \mathbf {x}')= \sum _{\sigma \in \mathscr {P}_k({\mathbf {x}})}p_t(\mathbf {x}, \hat{\tau }_z \mathbf {x}^{(\sigma )}) \end{aligned}$$

With a slight abuse of notation we denote by

39 $$\begin{aligned} X_N(\mathbf {x},\eta ,\varphi ):= \sum _{z\in \mathbb {Z}^d} \varphi \left( \frac{z}{N}\right) D(\hat{\tau }_z \mathbf {x},\eta ), \end{aligned}$$

define the *k*-th order fluctuation field associated to the *k*-particles configuration $\mathbf {x}$. Then we have

#### Theorem 3.1

Let $k:=||\mathbf {x}||$, then the *k*-th order fluctuation field $X_N(\mathbf {x},\eta ,\varphi )$ is such that

1. For all $$t>0$$
  40 $$\begin{aligned}&\mathbb {E}_{\nu _{\bar{\rho }}}\left[ X_N(\mathbf {x},\eta (t),\varphi )X_N(\mathbf {x},\eta (0),\varphi )\right] \nonumber \\&\quad = a(\xi (\mathbf {x}))\sum _{\sigma \in \mathscr {P}_k({\mathbf {x}})} \sum _{y,z\in \mathbb {Z}^d} \varphi \left( \frac{y}{N}\right) \varphi \left( \frac{z}{N}\right) p_t( \mathbf {x}, \hat{\tau }_{z-y} \mathbf {x}^{(\sigma )}) \end{aligned}$$
2. As a consequence, for $t>0$
  41 $$\begin{aligned}&\lim _{N\rightarrow \infty } N^{d(k-2)}\mathbb {E}_{\nu _{\bar{\rho }}}\left[ X_N(\mathbf {x},\eta (N^2t),\varphi )X_N(\mathbf {x},\eta (0),\varphi )\right] \nonumber \\&\quad = |\mathscr {P}_k(\mathbf {x})| a(\xi (\mathbf {x})) \frac{ d^{k/2}}{(2\pi t)^{dk/2}}\int _{\mathbb {R}^{2d}} e^{-kd|z-y|^2/2t} \varphi (z)\varphi (y) dz dy \end{aligned}$$

#### Proof

The first statement of the theorem is a direct application of Lemma 2.1 and the fact that the function $a(\cdot )$ is translation invariant, i.e. $a(\xi (\hat{\tau }_z \mathbf {x}))=a(\xi (\mathbf {x}))$, for all $z \in \mathbb {Z}^d$.

42 $$\begin{aligned}&\mathbb {E}_{\nu _{\bar{\rho }}}\left[ X_N(\mathbf {x},\eta (t),\varphi )X_N(\mathbf {x},\eta (0),\varphi )\right] \nonumber \\&\qquad \qquad = a(\xi (\mathbf {x}))\sum _{y,z\in \mathbb {Z}^d} \varphi \left( \frac{y}{N}\right) \varphi \left( \frac{z}{N}\right) p_t(\xi (\hat{\tau }_y \mathbf {x}), \xi (\hat{\tau }_z \mathbf {x})) \end{aligned}$$

Then, from (33) and (42) it follows that

43 $$\begin{aligned}&\mathbb {E}_{\nu _{\bar{\rho }}}\left[ X_N(\mathbf {x},\eta (t),\varphi )X_N(\mathbf {x},\eta (0),\varphi )\right] \nonumber \\&\qquad \qquad = a(\xi (\mathbf {x}))\sum _{\sigma \in \mathscr {P}_k({\mathbf {x}})} \sum _{y,z\in \mathbb {Z}^d} \varphi \left( \frac{y}{N}\right) \varphi \left( \frac{z}{N}\right) p_t( \mathbf {x}, \hat{\tau }_{z-y} \mathbf {x}^{(\sigma )}) \end{aligned}$$

For the second stament observe that from translation invariance we have

44 $$\begin{aligned} p^{\text {IRW}}_{N^2t}( \mathbf {x}, \hat{\tau }_{z-y} \mathbf {x})=\left( p_{N^2t}^{RW}(z-y)\right) ^k \end{aligned}$$

Define $B_{M,N}:=\{x\in \mathbb {Z}^d: |x|\le N M\}$, then, since $\varphi$ has a finite support we have that there exists $M\ge 0$ such that, for

$$\begin{aligned}&\sum _{y,z\in \mathbb {Z}^d} \varphi \left( \frac{y}{N}\right) \varphi \left( \frac{z}{N}\right) p^{\text {IRW}}_{N^2t}( \mathbf {x}, \hat{\tau }_{z-y} \mathbf {x})\\&\qquad \qquad =\sum _{y,z\in B_{M,N}} \varphi \left( \frac{y}{N}\right) \varphi \left( \frac{z}{N}\right) \left( p_{N^2t}^{RW}(z-y)\right) ^k\\&\qquad \qquad =\left( \frac{\sqrt{d}}{(2\pi t)^{d/2}}\right) ^k\left( 1+\frac{c}{N\sqrt{t}}\right) ^k\frac{1}{N^{kd}}\sum _{y,z\in B_{M,N}} \varphi \left( \frac{y}{N}\right) \varphi \left( \frac{z}{N}\right) \, e^{-\frac{kd|\frac{z}{N}-\frac{y}{N}|^2}{2t}} \end{aligned}$$

for a suitable $c=c(M)$, the last inequality coming from Theorem 6.2. We have

$$\begin{aligned}&\lim _{N\rightarrow \infty } \frac{1}{N^{2d}}\sum _{y,z\in \mathbb {Z}^d} \varphi \left( \frac{y}{N}\right) \varphi \left( \frac{z}{N}\right) e^{-\frac{kd|\frac{z}{N}-\frac{y}{N}|^2}{2t}}= \int _{\mathbb {R}^{2d}} \varphi \left( y\right) \varphi \left( z\right) \, e^{-\frac{kd|z -y|^2}{2t}} dxdz. \end{aligned}$$

$$\square $$

#### Quantitative Boltzmann–Gibbs Principle

On the same spirit than Corollary 3.1 we can now state a refined quantitative version of the Boltzmann–Gibbs principle for higher order fields.

##### Theorem 3.2

The field $X_N^{(k)}(\eta (N^ 2 t);\varphi )$ is such that for all $T>0$ there exists *C*(*T*) such that for all *N* big enough

45 $$\begin{aligned} \frac{1}{N^d}\int _0^T\int _0^T\mathbb {E}_{\nu _{\bar{\rho }}}\left[ X_N(\mathbf {x},\eta (N^2 t),\varphi )X_N(\mathbf {x},\eta (N^2 s),\varphi ) \right] \, ds \, dt \le C(T) N^{-\frac{2(k-1) d}{ 2 +(k-1) d}} \end{aligned}$$

##### Proof

Analogously to the case of two particles ( see the proof of Corollary 3.1), and using observation (44) we first obtain the following estimate

46 $$\begin{aligned}&\frac{1}{N^d} \mathbb {E}_{\nu _{\bar{\rho }}}\left[ X_N(\mathbf {x},\eta (N^2 t),\varphi )X_N(\mathbf {x},\eta (N^2 s),\varphi ) \right] \nonumber \\&\qquad \qquad \le \left( p_{N^2(t-s)}^{RW}(0)\right) ^{k-1} |\mathscr {P}_k(\mathbf {x})| a(\xi (\mathbf {x})) \Vert \varphi \Vert _1 \Vert \varphi \Vert _{\infty } \end{aligned}$$

again, by the LCLT

47 $$\begin{aligned} \left( p_{N^2(t-s)}^{RW}(0)\right) ^{k-1} \le {\left\{ \begin{array}{ll} \frac{d}{N^{(k-1)d} \epsilon _N^{(k-1)d/2}}, &{} \text {if}\ |t-s| \ge \epsilon _N \\ 1, &{} \text {otherwise} \end{array}\right. } \end{aligned}$$

allowing us to bound the integral

48 $$\begin{aligned}&\int _0^T\int _0^T \frac{1}{N^d}\mathbb {E}_{\nu _{\bar{\rho }}}\left[ X_N^{(2)}(\eta (N^ 2 t);\varphi ) X_N^{(2)}(\eta (N^2 s);\varphi )\right] \, ds \, dt \nonumber \\&\qquad \qquad \qquad \le |\mathscr {P}_k(\mathbf {x})| a(\xi (\mathbf {x})) \Vert \varphi \Vert _1 \Vert \varphi \Vert _{\infty } \frac{T^2}{2} \left[ \frac{d}{N^{(k-1)d} \epsilon _N^{(k-1)d/2}} + d \epsilon _N \right] \end{aligned}$$

the same anzats, $\epsilon _N = N^{-\alpha }$, results on the optimal value

49 $$\begin{aligned} \alpha = \frac{2(k-1) d}{ 2 +(k-1) d}. \end{aligned}$$

$$\square $$

### Fluctuation Fields of Projections on ${\mathscr {H}}_N$

We can further generalize part (2) of Theorem 3.1 to a wider class of functions *f*. In this section we make such a generalization for a particular subset of $L^2(\nu _\rho )$. For $f\in L^2(\nu _\rho )$ we can use the fact that the union of the spaces ${\mathscr {H}}_n$ is dense in $L^2(\nu _\rho )$ to express *f* as follows

50 $$\begin{aligned} f (\eta ) = \sum _{\begin{array}{c} n \ge 0 \\ \xi \in \Omega _f: \Vert \xi \Vert = n \end{array}} C_{n,\xi } D(\xi ,\eta ) \end{aligned}$$

for the rest of this section we restrict ourselves to the set of functions $f\in L^2(\nu _\rho )$ satisfying the following condition

51 $$\begin{aligned} \sum _{ \xi , \xi ^\prime \in \Omega _f: \Vert \xi \Vert = \Vert \xi ^\prime \Vert } | C_{n,\xi } C_{n,\xi ^\prime } | a(\xi ^\prime ) < \infty \end{aligned}$$

In particular all linear combinations of orthogonal duality polynomials satisfy (51).

#### Theorem 3.3

Let *f* be a function such that the condition (51) is satisfied, and as before let $f_{k-1}$ denote the projection of *f* on ${\mathscr {H}}_{k-1}$, then the field

$$\begin{aligned} \mathsf {X_N}(f-f_{k-1},\eta ;\varphi )=\sum _{x\in \mathbb {Z}^d} (\tau _x f (\eta )- \tau _x f_{k-1}(\eta )) \varphi \left( \frac{x}{N}\right) \end{aligned}$$

satisfies

$$\begin{aligned} \mathbb {E}_{\nu _{\bar{\rho }}}\left[ \mathsf {X_N}(f-f_{k-1},\eta ;\varphi )\mathsf {X_N}(f-f_{k-1},\eta (N^2 t);\varphi )\right] = O(N^{-d(k-2)}) \end{aligned}$$

#### Proof

After some simplifications due to orthogonality the field reads

$$\begin{aligned} \mathsf {X_N}(f-f_{k-1},\eta ;\varphi )= \sum _{x \in \mathbb {Z}^d} \varphi \left( \frac{x}{N}\right) \sum _{\begin{array}{c} n \ge k \\ \xi \in \Omega _f: \Vert \xi \Vert = n \end{array}} C_{n,\xi } \tau _x D( \xi ,\eta ) \end{aligned}$$

We then compute

52 $$\begin{aligned}&\mathbb {E}_{\nu _{\bar{\rho }}}\left[ \mathsf {X_N}(f-f_{k-1},\eta ;\varphi )\mathsf {X_N}(f-f_{k-1},\eta (N^2 t);\varphi )\right] \nonumber \\&\quad = \sum _{x, y} \varphi \left( \frac{x}{N}\right) \varphi \left( \frac{y}{N}\right) \sum _{\begin{array}{c} n \ge k \\ \xi \in \Omega _f: \Vert \xi \Vert = n \end{array}} \sum _{\begin{array}{c} l \ge k \\ \xi ^\prime \in \Omega _f: \Vert \xi ^\prime \Vert = l \end{array}} C_{n,\xi } C_{l,\xi ^\prime }\nonumber \\&\qquad \int \tau _x D(\xi ,\eta ) E_\eta \left[ \tau _y D(\xi ^\prime ,\eta (N^2 t)) \right] d \nu _{\bar{\rho }} (\eta ) \nonumber \\&\quad = \sum _{x, y} \varphi \left( \frac{x}{N}\right) \varphi \left( \frac{y}{N}\right) \sum _{\begin{array}{c} n \ge k \\ \xi \in \Omega _f: \Vert \xi \Vert = n \end{array}} \sum _{\begin{array}{c} l \ge k \\ \xi ^\prime \in \Omega _f: \Vert \xi ^\prime \Vert = l \end{array}} C_{n,\xi } C_{l,\xi ^\prime } \nonumber \\&\qquad \int \tau _x D(\xi ,\eta ) E_\eta \left[ \tau _y D(\xi ^\prime ,\eta (N^2 t)) \right] d \nu _{\bar{\rho }} (\eta ) \nonumber \\&\quad = \sum _{x, y} \varphi \left( \frac{x}{N}\right) \varphi \left( \frac{y}{N}\right) \sum _{\begin{array}{c} n \ge k \\ \xi \in \Omega _f: \Vert \xi \Vert = n \\ \xi ^\prime \in \Omega _f: \Vert \xi ^\prime \Vert = n \end{array}} C_{n,\xi } C_{n,\xi ^\prime } a(\xi ^\prime ) p_{N^2 t}(\tau _y \xi ^\prime , \tau _x \xi ) \end{aligned}$$

from the LCLT we can also obtain that

$$\begin{aligned} p_{N^2 t}(\tau _y \xi , \tau _x \xi ^\prime ) = \mathscr {O}(N^{-d \Vert \xi \Vert }) \end{aligned}$$

this, allows us to bound our expression of interest

53 $$\begin{aligned}&N^{d(k-2)} \mathbb {E}_{\nu _{\bar{\rho }}}\left[ \mathsf {X_N}(f-f_{k-1},\eta ;\varphi )\mathsf {X_N}(f-f_{k-1},\eta (N^2 t);\varphi )\right] \nonumber \\&\qquad \qquad \le N^{d(k-2)} \sum _{x, y} \varphi \left( \frac{x}{N}\right) \varphi \left( \frac{y}{N}\right) \sum _{\begin{array}{c} n \ge k \\ \xi \in \Omega _f: \Vert \xi \Vert = n \\ \xi ^\prime \in \Omega _f: \Vert \xi ^\prime \Vert = n \end{array}} \frac{M}{N^{dn}} | C_{n,\xi } C_{n,\xi ^\prime }| a(\xi ^\prime ) \nonumber \\&\qquad \qquad = \left( \frac{1}{N^{2d}}\sum _{x, y} \varphi \left( \frac{x}{N}\right) \varphi \left( \frac{y}{N}\right) \right) \sum _{\begin{array}{c} n \ge k \\ \xi \in \Omega _f: \Vert \xi \Vert = n \\ \xi ^\prime \in \Omega _f: \Vert \xi ^\prime \Vert = n \end{array}} \frac{M}{N^{d(n-k)}} | C_{n,\xi } C_{n,\xi ^\prime }| a(\xi ^\prime ) \end{aligned}$$

At this point we need to show that the last summation does not play a role in the leading order. But this comes from the fact that *f* satisfies condition (51). $\square$

Analogously to Theorem 3.2 we provide a quantitative version of the Boltzmann–Gibbs principle for the current setting.

#### Theorem 3.4

The field $\mathsf {X_N}(f-f_{k-1},\eta ;\varphi )$ is such that for all $T>0$ there exists *C*(*T*) such that for all *N* big enough

54 $$\begin{aligned}&\frac{1}{N^d}\int _0^T\int _0^T\mathbb {E}_{\nu _{\bar{\rho }}}\left[ \mathsf {X_N}(f-f_{k-1},\eta (N^2 t);\varphi ) \mathsf {X_N}(f-f_{k-1},\eta (N^2 s);\varphi ) \right] \, ds \, dt\nonumber \\&\qquad \qquad \qquad \le C(T) N^{-\frac{2(k-1) d}{ 2 +(k-1) d}}. \end{aligned}$$

## Non-stationary Fluctuation Fields

### Second Order Fields

Let us now start independent walkers from a product measure of non-homogeneous Poisson, with weakly varying density profile i.e., from the measure $\nu _{\bar{\rho }}=\otimes _{x\in \mathbb {Z}^d} \nu _{\rho (x)}$ where $\bar{\rho } \in \mathbb {R}^{\mathbb {Z}^d}$ and $\rho (x)$ is given by the relation $\bar{\rho } = (\rho (x))_{x \in \mathbb {Z}^d}$ . We denote by  the orthogonal polynomials, i.e.,where  denote the orthogonal polynomials w.r.t. Poisson with parameter $\rho (i)$.

We also denote by $\bar{\rho }_t = (\rho (x))_{x \in \mathbb {Z}^d}$, where $\rho _t(x)= \mathbb {E}_x\left[ \rho (X_t)\right]$ and $X_t$ denotes the continuous-time random walk. We now are interested in the fields55then the second order field is56with respect to previous notation please notice the additional dependence on the parameter $\bar{\rho }$ and in time *t*.

We want to prove that the covariance of $X_N^{(2)}(\bar{\rho },\varphi ,t)$ and $X_N^{(2)}(\bar{\rho },\varphi ,s)$ is of order 1, as $N\rightarrow \infty$, exactly as in the stationary case. For this we start with the following result:

#### Lemma 4.1

Let $\nu _{\bar{\rho }}:=\otimes _{x\in \mathbb {Z}^d} \nu _{\rho (x)}$ be a product of non-homogeneous Poisson measures, then we have57where


#### Proof

Note thathence58We now state the following:

**Claim 1:**Indeed, by duality, $\mathbb {E}_\eta \left[ \eta _x(t)-\rho _t(x)\right] =\sum _z p_t(x,z) (\eta _z-\rho (z))$ and $(\eta _z-\rho (z))$ is in $L^2(\nu _{\bar{\rho }} (\eta ))$ always orthogonal to  because for $z\ne y$ both $(\eta _z-\rho (z))$ and  have expectation zero and when $z=y$ because it is the inner product of the first order and second order orthogonal polynomials, which is zero. So we only have to work out the expectation $\mathbb {E}_\eta \left[ \eta _x(t)(\eta _x(t)-1)\right]$ which by duality equals

$$\begin{aligned} \sum _{u} p_t(x,u)^2 \eta _u(\eta _u-1) + 2\sum _{u\not = v}p_t(x,u) p_t(x,v) \eta _u \eta _v \end{aligned}$$

**Claim 2:** For all *u*Indeed, for $u\not = y$ this is true because of the product character of the measure and the fact that  has zero expectation, and for $u=y$ $\eta _y=\eta _y-\rho (y) + \rho (y)$ which is the sum of the first orthogonal polynomial and a constant, which is in $L^2(\nu _{\bar{\rho }} (\eta ))$ orthogonal to .

Finally, we remark that for all $u\not =y$because of the product character of the measure and the fact that  has zero expectation. Finally,because adding first order terms in $\eta _y$ does not change the inner product with .

$$\square $$

As a consequence of Lemma 4.1 and using that a product of Poisson measures is reproduced at later times, we compute

59 $$\begin{aligned}&\lim _{N\rightarrow \infty }\mathbb {E}_{\nu _{\bar{\rho }}} \left[ X_N^{(2)}(\bar{\rho },\varphi ,t) X_N^{(2)}(\bar{\rho }, \varphi , s)\right] \nonumber \\&\qquad \qquad = \lim _{N\rightarrow \infty }\mathbb {E}_{\nu _{{\bar{\rho }}_{sN^2}}} \left[ X_N^{(2)}(\bar{\rho },\varphi ,t-s) X_N^{(2)}(\bar{\rho }, \varphi , 0)\right] \nonumber \\&\qquad \qquad = \int \frac{e^{-\frac{(x-y)^2}{t-s}}}{2\pi (t-s)^{d/2}}\; \varphi (x)\varphi (y) \, \kappa _2(y) dx dy \end{aligned}$$

where

$$\begin{aligned} \kappa _2(y)= \lim _{N\rightarrow \infty } k_2(Ny) \end{aligned}$$

which exists because the initial Poisson measure has slowly varying density profile.

### Higher Order Fields: Non-stationary Case

The aim of this section is to extend the results of the previous example to higher order fields:60We start then with a generalization of Lemma 4.1 to higher orders. As we already stated in Remark 2.2 in the case of independent random walkers, the orthogonal duality polynomials are related to the classical duality polynomials in the following way:61where *d*(*k*, *n*) are the classical single site duality polynomials.

#### Remark 4.1

Notice that due to the non-homogeneity of the product measure, the duality property cannot be any longer guaranteed.

Despite of the previous remark, the special form of the Charlier polynomials allows us to reach the same conclusions than in the stationary case. Let us first make a simple observation:

Define $A(\xi , \eta ,\bar{\rho })$ as the difference between the Charlier and classical polynomials of order $\Vert \xi \Vert$, i.e.and notice that $A(\xi , \eta ,\bar{\rho })$ is a polynomial of degree strictly less than $\Vert \xi \Vert$ and as a consequence it has an expansion, in terms of orthogonal polynomials, consisting only on polynomials of order strictly smaller than $\Vert \xi \Vert$. Therefore, by orthogonality we havefor any configuration $\xi ^\prime$ such that $\Vert \xi \Vert \le \Vert \xi ^\prime \Vert$. With this observation we are ready to state the following Lemma:

#### Lemma 4.2

Let $\nu _{\bar{\rho }}:=\otimes _{x\in \mathbb {Z}^d} \nu _{\rho (x)}$ be a product of non-homogeneous Poisson measures, and let $\rho _t(x)= \mathbb {E}_x\left[ \rho (X_t)\right]$, where $X_t$ denotes continuous-time random walk. Then we have62where $.$

#### Proof

We simply compute63where in the fourth and fifth line we subtracted and added zero respectively by using the orthogonality of  to lower order polynomials in the expansion. $\square$

We now state the non-stationary version of Theorem 3.1.

#### Theorem 4.1

Let $\nu _{\bar{\rho }}:=\otimes _{x\in \mathbb {Z}^d} \nu _{\rho (x)}$ and $\rho _t(x)$ be as before, and let $k:=||\mathbf {x}||$, then

1. For all $$t>0$$
  64 $$\begin{aligned}&\mathbb {E}_{\nu _{\bar{\rho }}} \left[ X_N(\mathbf {x},\bar{\rho },\varphi ,t) X_N(\mathbf {x} ,\bar{\rho },\varphi ,0)\right] \nonumber \\&\quad = a_0\left( \sum _{i=1}^k\delta _{x_i} \right) \sum _{x,y} \varphi (\tfrac{x}{N})\varphi (\tfrac{y}{N}) p_t \left( \sum _{i=1}^k\delta _{ x+x_i}; \sum _{i=1}^k \delta _{y+x_i}\right) \end{aligned}$$
2. As a consequence, for $t>s>0$
  $$\begin{aligned}&\lim _{N\rightarrow \infty } N^{d(k-2)}\mathbb {E}_{\nu _{\bar{\rho }}} \left[ X _N(\mathbf {x},\bar{\rho },\varphi ,t) X_N(\mathbf {x},\bar{\rho },\varphi ,s) \right] \\&\quad = {\mathscr {K}}(x_1,\ldots , x_k;\rho ) \frac{d^{k/2}}{(2\pi (t-s))^{dk/2}}\int _{\mathbb {R}^2} e^{-kd(x-y)^2/2(t-s)} \varphi (x)\varphi (y) dx dy \end{aligned}$$
  with $\xi = \sum _{i=1}^k\delta _{x_i}$ and ${\mathscr {K}}(x_1,\ldots , x_k;\rho )$ defined as in the stationary case.

#### Proof

Is a consequence of Lemma 4.2 together with the fact that a product of Poisson measure is reproduced at later times. $\square$

With this last theorem, we have now the ingredients to obtain a quantitative Boltzmann–Gibbs principle.

#### Corollary 4.1

For all $T>0$ there exists *C*(*T*) such that for all *N* big enough

65 $$\begin{aligned} \frac{1}{N^d}\int _0^T\int _0^T \mathbb {E}_{\nu _{\bar{\rho }}} \left[ X _N(\mathbf {x},\bar{\rho },\varphi ,t) X _N(\mathbf {x},\bar{\rho },\varphi ,s) \right] \, ds \, dt \le C(T) N^{-\frac{2(k-1) d}{ 2 +(k-1) d}} \end{aligned}$$

#### Proof

The proof is essentially the same than in all the previous cases. $\square$

## Particle Systems with Orthogonal Duality

In the context of stationarity, the results of this paper are not exclusive for independent random walkers. Hence in this section we extend our results to a wider class of IPS. i.e. to those particle systems that enjoy the existence of orthogonal self-duality and that satisfy an additional condition in the transition kernel. Let then $\{ \eta _t \}_{ t \ge 0}$ be an IPS for which there exists an orthogonal self-duality function $D: \Omega _f \times \Omega \rightarrow \mathbb {R}$ satisfying all the properties stated in Sect. 2.2. As in the same section, we denote by $p_t(\xi ,\xi ^\prime )$ the transition probability to go from configuration $\xi$ to $\xi ^\prime$ in time *t*. Then, immediately follows the following:

### Lemma 5.1

Let $$\xi ,\xi '\in \Omega _f$$, then

66 $$\begin{aligned} \int \mathbb {E}_\eta (D(\xi ,\eta _t)) D(\xi ',\eta ) d\nu _{\bar{\rho }}(\eta )= p_t(\xi ,\xi ') a(\xi ') \end{aligned}$$

furthermore, let us assume that for all $\xi ,\xi '\in \Omega _f$, the transition kernel satisfies the following estimate

67 $$\begin{aligned} p_t (\xi , \xi ^\prime ) \le \frac{C}{(1+t)^{ \Vert \xi \Vert d/2 }} \end{aligned}$$

This assumption is reasonable, since in [9] estimates of this kind were already found for a wide class of interacting particle systems that for example includes generalized exclusion processes. The results of [9] are applicable as long as the process satisfies a logarithmic Sobolev inequality for the symmetric part of the generator. As before, for a fix $\mathbf {x} \in \mathbb {Z}^{dk}$ we define the polynomial fluctuation field

68 $$\begin{aligned} X_N(\mathbf {x},\eta ,\varphi ):= \sum _{z\in \mathbb {Z}^d} \varphi \left( \frac{z}{N}\right) D(\hat{\tau }_z \mathbf {x},\eta ), \end{aligned}$$

from assumption (67) we can also conclude.

### Theorem 5.1

For all $T>0$ there exists *C*(*T*) such that for all $x \in \mathbb {Z}^{dk}$ and for all *N* big enough

69 $$\begin{aligned} \frac{1}{N^d}\int _0^T\int _0^T\mathbb {E}_{\nu _{\bar{\rho }}}\left[ X_N(\mathbf {x},\eta (N^2 t),\varphi )X_N(\mathbf {x},\eta (N^2 s),\varphi ) \right] \, ds \, dt \le C(T) N^{-\frac{2(k-1) d}{ 2 +(k-1) d}} \end{aligned}$$

## Appendix

### Local Limit Theorems

In this section we state and prove a local central limit theorem for independent random walkers in continuous time. The motivation of this section comes from the fact that, despite of being common knowledge, we were not able to find a reference that includes the proof of such a result. However we do have access to many versions of the discrete case. We state now the version included in [10], since we consider is the most suitable to then jump to the continuous time case. Theorem 6.1 below is a direct consequence of Theorem 2.1.1 in the same reference [10].

#### Theorem 6.1

(LCLT for discrete-time random walk) Let $x\in \mathbb {Z}^d$ and $p^{\text {DRW}}_n(\cdot )$ be the probability distribution of a discrete-time random walk in $\mathbb {Z}^d$, then, for any fixed $M\ge 0$ there exists $c=c(M)$ such that

70 $$\begin{aligned} \sup _{|x|\le M\sqrt{n}}\bigg |\frac{p^{\text {DRW}}_n(x)}{\bar{p}_n(x)}-1 \bigg | \le \frac{c}{n} \end{aligned}$$

where

71 $$\begin{aligned} \bar{p}_t(x):=\frac{\sqrt{d}}{(2\pi t)^{d/2}}\, e^{-\frac{d|x|^2}{2t}} \end{aligned}$$

The way we generalize this theorem is by means of the following.

#### Theorem 6.2

(LCLT for continuous-time random walk) Let $x\in \mathbb {Z}^d$ and $p^{\text {RW}}_t(\cdot )$ be the probability distribution of a continuous-time random walk in $\mathbb {Z}^d$, then, for any fixed $M\ge 0$ there exists $c=c(M)>0$ s.t.

72 $$\begin{aligned} \sup _{|x|\le M \sqrt{t}} \bigg | \frac{p^{RW}_t(x)}{\bar{p}_t(x)}-1\bigg | \le \frac{c}{\sqrt{t}} \end{aligned}$$

#### Proof

We can always decompose

73 $$\begin{aligned} p^{\text {RW}}_t(x)=\sum _{n=0}^\infty P(N_t=n) \, p^{\text {DRW}}_n(x) \end{aligned}$$

with $N_t$ a Poisson process of rate 1. First by Proposition 2.5.5 in [10] we have

74 $$\begin{aligned} P(N_t=n)=\frac{1}{\sqrt{2\pi t}}\, e^{-\frac{(n-t)^2}{2t}} \, \exp \left\{ \mathscr {O}\left( \frac{1}{\sqrt{t}}+\frac{|n-t|^3}{t^2}\right) \right\} \end{aligned}$$

Now for $\epsilon >0$, we assume that

$$\begin{aligned} \frac{|n-t|}{t} \le \epsilon \end{aligned}$$

after some manipulation we obtain the following relations

75 $$\begin{aligned} \frac{1}{n} = \frac{1}{t} \left( 1+\mathscr {O}\left( \frac{|n-t|}{t}\right) \right) , \qquad \frac{1}{ n^{\alpha }} = \frac{1}{t^{\alpha }} \left( 1+\mathscr {O}\left( \frac{|n-t|}{t}\right) \right) \end{aligned}$$

combining (75) with Theorem 6.1 we have

76 $$\begin{aligned} p^{\text {DRW}}_n(x)= & {} \frac{\sqrt{d}}{(2\pi n)^{d/2}}\, e^{-\frac{d|x|^2}{2n}} \left( 1+\mathscr {O}\left( \frac{1}{n}\right) \right) \nonumber \\= & {} \frac{\sqrt{d}}{(2\pi t)^{d/2}}\, e^{-\frac{d|x|^2}{2n}} \exp \left\{ \mathscr {O}\left( \frac{|x|^2|n-t|}{t^2}\right) \right\} \left( 1+\mathscr {O}\left( \frac{1}{t}\right) \right) \left( 1+\mathscr {O}\left( \frac{|n-t|}{t}\right) \right) \nonumber \\ \end{aligned}$$

Finally, substitution of (74) and (76) in (73) and further manipulations gives

77 $$\begin{aligned}&\sum _{n=0}^\infty P(N_t=n) p^{\text {DRW}}_n(x) \nonumber \\&\qquad =\sum _{n=0}^\infty \frac{1}{\sqrt{2\pi t}}\, e^{-\frac{(n-t)^2}{2t}} \, \exp \left\{ \mathscr {O}\left( \frac{1}{\sqrt{t}}+\frac{|n-t|^3}{t^2}\right) \right\} \; \nonumber \\&\qquad \quad \times \,\frac{\sqrt{d}}{(2\pi t)^{d/2}}\, e^{-\frac{d|x|^2}{2t}}\, \exp \left\{ \mathscr {O}\left( \frac{|x|^2|n-t|}{t^2}\right) \right\} \left( 1+\mathscr {O}\left( \frac{1}{t}\right) \right) \left( 1+\mathscr {O}\left( \frac{|n-t|}{t}\right) \right) \nonumber \\ \end{aligned}$$

Assuming $|x|\le M \sqrt{t}$ and using (6.1), we get the following,

78 $$\begin{aligned} \exp \left\{ \mathscr {O}\left( \frac{|x|^2|n-t|}{t^2}\right) \right\} = \exp \left\{ \mathscr {O}\left( \epsilon \right) \right\} \end{aligned}$$

Hence, more applications of (6.1) give

$$\begin{aligned}&\sum _{n=0}^\infty P(N_t=n) p^{\text {DRW}}_n(x) \\&\qquad \qquad = \left( 1+\mathscr {O}\left( \frac{1}{t}\right) \right) \frac{\sqrt{d}}{(2\pi t)^{d/2}} e^{-\frac{d|x|^2}{2t}} \exp \left\{ \mathscr {O}\left( \epsilon \right) \right\} \left( 1+\mathscr {O}\left( \epsilon \right) \right) \exp \left\{ \mathscr {O}\left( \frac{1}{\sqrt{t}}\right) \right\} \\&\qquad \qquad \quad \times \sum _{n=0}^\infty \frac{1}{\sqrt{2\pi t}}\, e^{-\frac{(n-t)^2}{2t}} \, \exp \left\{ \mathscr {O}\left( \frac{|n-t|^3}{t^2}\right) \right\} \; \\&\qquad \qquad = \bar{p}_t(x)\left( 1+\mathscr {O}\left( \frac{1}{\sqrt{t}}\right) \right) \end{aligned}$$

$$\square $$
